# Environmental Factors for Sustained Telehealth Use in Mental Health Services: A Mixed Methods Analysis

**DOI:** 10.1155/2024/8835933

**Published:** 2024-09-16

**Authors:** Benjamin Werkmeister, Anne M. Haase, Theresa Fleming, Tara N. Officer

**Affiliations:** ^1^ School of Health Te Herenga Waka-Victoria University of Wellington, Wellington, New Zealand; ^2^ Department of Psychological Medicine Te Whatu Ora-Health New Zealand, Wellington, New Zealand; ^3^ Department of Psychological Medicine University of Otago-Wellington, Wellington, New Zealand; ^4^ School of Nursing Midwifery and Health Practice Te Herenga Waka-Victoria University of Wellington, Wellington, New Zealand

**Keywords:** clinician, interpretive description, mental health services, mixed methods research, telehealth

## Abstract

**Background:** The mental health service delivery gap remains high globally. Appropriate telehealth use may increase capacity through flexible remote care provision. Despite the historical lack of telehealth integration into publicly funded mental health services, during COVID-19 lockdowns, services rapidly switched to telephone and audiovisual care provision. In Aotearoa New Zealand (NZ), this was abandoned when no longer required by COVID-19 restrictions. This study explores environmental factors associated with telehealth implementation and ongoing use or discontinuation across a multiregional outpatient mental health service. This work contributes to understanding system-level factors influencing telehealth use and thus informs policy and practice in postpandemic environments.

**Methods:** This mixed methods study applied an interpretive description methodology. Semistructured interviews with 33 mental health clinicians were thematically analysed. Qualitative findings were reframed and evaluated using time series analyses of population-level quantitative data (prior to and throughout the pandemic). Findings were synthesised with qualitative themes to develop an understanding of environmental factors contributing to telehealth use.

**Results:** Findings highlighted an increase in clients assessed by mental health services and declining clinician numbers, contributing to pressure placed on clinicians. There was a lack of culture supporting telehealth, including limited awareness, leadership, and champions to facilitate implementation. Some teams provided services suited to telehealth; other subspeciality teams had limited applications for telehealth. There was a general lack of policy and guidelines to support telehealth use and limited technical support for clinicians unfamiliar with audiovisual software.

**Conclusion:** Disorganised telehealth adoption in the study regions provides insight into wider environmental drivers affecting telehealth uptake. For telehealth to become a workable service delivery mode following COVID-19, stewardship and culture shifts are required, including policy development, technical support, and resources to support clinical teams. Telehealth may address growing service demand by improving interfaces with primary care and providing timely access to specialist input.

## 1. Introduction

Internationally, there is a growing and exigent demand for specialist mental health and addiction services, with a 25% rise in prevalence of mental health conditions from 2000 to 2019 [1], with approximately 970 million people having a mental disorder in 2019, before the COVID-19 pandemic. Mental illness creates a significant burden on the global economy, and yet, the median government health expenditure for mental health services comprises only 2% of the total health expenditure [1].

In Aotearoa New Zealand (NZ), there has been a 23% rise in clients accessing specialist mental health services from 2006 to 2017 [2, 3], with a corresponding 40% increase in public funding [3]. Approximately 5% of New Zealanders report an unmet need for mental health treatment [4], and national targets for timely access to specialist mental healthcare are not being met [2]. The NZ mental health workforce faces resource constraints, capacity limitations, burnout, and growing service demand [5]. Mental health workforce staffing has not kept pace with population growth, due to the limited future planning, a high reliance on overseas-trained health professionals, and poor staff retention [3]. Increased workforce pressure may increase burnout, which is an issue reported extensively internationally [6–8].

The COVID-19 pandemic prompted health system adaptation to minimise virus transmission. Telehealth (health services provided via telephone and audiovisual videoconferencing) facilitated synchronous care delivery from a distance, resulting in a surge in telehealth delivery within outpatient mental health services [9, 10]. Internationally, many health services are now working towards sustainable implementation of telehealth as an alternative and complement to in-person mental health service provision [11, 12]. Such initiatives are particularly valuable given pandemic-related stressors, increased prevalence of severe mental health disorders/service demand [13–15], and concerns around the emergence of increased mental health service demand postpandemic [14, 16]. However, in NZ, telehealth provision receded when social distancing was no longer required [17, 18].

Telehealth is cost-effective [19] and can be used to improve service delivery [20] and expand mental healthcare access to rural and underresourced regions [21–23]. Telehealth can be used to conduct mental health assessments, provide treatment [24–26], and maintain therapeutic relationships with clients [27]. However, there is limited understanding of service pressures and clinical needs at a health system level that would support the integration of telehealth into mental healthcare delivery.

Much of what does exist in the implementation space focuses on single-centre qualitative studies [28–33]. Notably, there also remains a gap when considering telehealth use in delivering services for populations with more severe mental health need and variable perspectives around the impact telehealth may have on therapeutic alliances in mental health [34–38]. However, there is a growing recognition of the potential for telehealth as a service delivery option [39–45], while also recognizing the potential for telehealth to perpetuate digital exclusion in mental health populations [38, 46, 47].

The rise and fall of telehealth during the COVID-19 pandemic in NZ provided a unique opportunity to explore (1) wider patterns of service delivery and (2) the health service ecosystem that contributed to changes in telehealth provision. Our prior work in this space focussed on the impact of COVID-19 lockdown on mental health clinicians [18], clinician factors that influenced telehealth uptake [48], and mental health client experiences of using telehealth during COVID-19 lockdown [17]. Findings from this work underscore particular issues with introducing and delivering telehealth services among a high-risk mental health population, including more unique issues around the capacity to consent and be involved in telehealth consultations [17] but also issues highlighting problems with clinician readiness and discernment of nonverbal cues [48]. The aim of the present work is to identify factors related to service provision trends and the healthcare environment that influenced telehealth implementation (during periods of stay-in-place/lockdown) and ongoing use within mental health services.

Focusing on mental health service delivery is particularly important given issues with condition severity, chronicity, and the potential wider social impacts of mental health conditions. Postpandemic, the present exploration could be used to inform sustainable implementation strategies for publicly funded outpatient specialist mental health services internationally and inform health system redesign, such as the restructuring of mental health services in NZ [49]. Given the recent recognition of the impacts lockdowns and other COVID-19 policies had on population wellbeing and healthcare engagement [50, 51], such work is timely.

## 2. Methods

**Figure 1:**
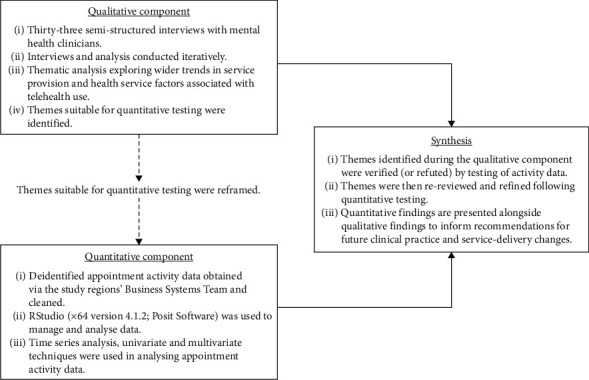
Methods overview.

This sequential mixed methods research applied an interpretive description methodology [52] to facilitate development of clinically relevant recommendations that inform health service delivery. This paper focuses on the nexus between COVID-19 responses, trends in mental healthcare in the study region, and environmental factors contributing to the uptake and withdrawal from telehealth provision. Broadly, methods (Figure 1) involved semistructured interviews with clinicians and the analysis of administrative activity data. These are outlined below; detailed methods have been previously published [18, 48].

### 2.1. Context

This study encompassed 22 publicly funded mental health outpatient teams across 3 NZ regions that work under 1 administrative umbrella. The teams included those providing adult, child, and older person care to all ethnic groups and culturally specific services tailored to Indigenous Māori populations that provided Kaupapa Māori (Māori approach, led by Māori clinicians) and Pacific populations that offer traditional culturally led healing-based care. Each team consisted of between 5 and 26 mental health clinicians. Clinicians from all but 1 of these 22 teams participated in interviews.

### 2.2. Methodological Context

First proposed by Thorne et al. [52], interpretive description follows principles of interpretivism, recognising that reality is not absolute and can be interpreted and is, therefore, complex [53]. Epistemologically, knowledge is seen as inseparable from the knower and is constructed.

Thorne [54] defines interpretive description as an approach requiring purpose derived from practice goals and an understanding of what is known based on available evidence. The disciplinary orientation of interpretive description is central to informing data analysis, as it facilitates a recognition of relevant data patterns [55] and an “interactive flow of ideas” ([56], p. 250) between research and practice that are challenged and refined as research is conducted [57]. This pragmatism means that such a methodology is well suited to mixed methods research [58] as quantitative research can help frame the credibility of claims made in individual interpretations [53] and in so doing support the identification of data patterns highlighted through qualitative interviews. The study's lead author (B.J.W.), a psychiatric medical registrar, has worked within several of these teams placing them as a research insider. Participants known to the lead author were interviewed by another member of the wider research team. A field log was kept, supporting triangulation of findings arising during thematic analysis; this log detailed interactions with participants, prior interaction with participants, and reflection on content of interviews. Transcript coding was independently reviewed by coauthors to improve the validity of the coding framework.

### 2.3. Qualitative Methods

Participants were identified through directly approaching outpatient team leaders and, in many cases, talking directly to outpatient teams to introduce the study. Thirty-five clinicians showed interest in research participation and 33 participated in semistructured one-to-one interviews (guided by a prepiloted interview schedule) between November 2020 and February 2021 in the study regions. This represents between one and three clinicians within each recruited outpatient team. Individuals were included in this study if they had delivered telehealth care during periods of lockdown over the pandemic and had been working in an outpatient team. Interviews took place in person or via videoconferencing software (Zoom) as mutually agreed between the participant and interviewer. As thanks for the participants' time, following interviews, outpatient teams received a morning/afternoon tea. Recruitment halted at 33 interviews; aligning with interpretive description principles [55], there was confidence that findings provided sufficient relevant variation and complexity to inform practice recommendations without new information emerging.

A third party, working under a confidentiality agreement, transcribed the interview recordings. Deidentified transcripts were checked by B.J.W. for completeness and uploaded to NVivo 12 (QSR International) for thematic analysis in line with principles outlined by Braun and Clarke [59]. Prior to uploading, 1 transcript was updated in response to a participant's review (11 participants in total requested copies of their transcript). Initial codes were developed based on clusters of shared meaning and then refined iteratively. Themes were reviewed within the wider research team, and differences and distinctions were discussed, in line with the principles of thematic analysis [59]. The wider research team acted as external critics [55] and triangulated findings with the wider project.

Participant demographic details were collected following interviews using a standard questionnaire. Participants were predominantly female (*n* = 23) and came from a range of ethnic groups, including Indigenous Māori (*n* = 5) and NZ European (*n* = 22). Ages ranged from 25 to 65+ years, with a modal age range of 45–54 years. Participants represented a wide variety of clinician roles (seven doctors, six nurses, five clinical psychologists, five team leaders, three social workers, two occupational therapists, one kaumātua [respected Māori elder], one cultural therapist, and one psychotherapist). To protect participants' privacy, demographic information has not been provided alongside participant quotes.

Themes relating to wider trends in mental health service provision and environmental factors influencing telehealth use are presented in this paper. Five of the eight themes that arose during analysis contained assertions that could be verified using quantitative testing.

### 2.4. Reframing Themes for Quantitative Testing

Qualitative themes can be triangulated and supported through quantitative testing, which is valuable for contextualising and enriching understandings of complex research questions [58, 60]. During thematic analysis, we identified themes related to trends in service demands and provision influencing telehealth use, which leant well to quantitative testing. The assertions contained within these themes were reframed (Table 1) to guide quantitative exploration of key aspects of service delivery through analysis of administrative data.

### 2.5. Quantitative Methods

**Figure 2:**
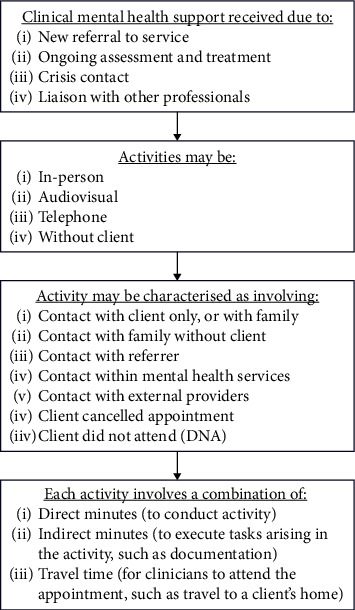
Activity flow for mental health services.

Outpatient activity data, used for auditing and recording clinician activities, was sourced and used for quantitative analysis. This activity data is the only available record of public specialist mental health appointments in the study region and outlines all activities linked to client–clinician appointments (conceptualised in Figure 2). It is manually produced following each clinical interaction around a client activity. Via the mental health services' business systems team, the research team sourced deidentified activity data between 2 September 2019 and 1 August 2022, representing over four million clinical activity observations. This data provided limited information on participant characteristics (client age, gender and ethnicity, and clinician role), appointment characteristics (duration, location, family involvement, mode of delivery and if telehealth was used, whether an appointment was cancelled/the client did not attend, and other indirect time related to an appointment), and date of referral and discharge. Modes of delivery documented in the data included AV, telephone, and in-person. Client demographic information was automatically populated when entering activity data.

On 21 March 2020, the NZ Government introduced a four-level alert system to mitigate COVID-19 transmission based on the extent of community exposure and risk of spread. The almost 3 years this data covered included two periods (23 March 2020 to 13 May 2020 and 17 August 2021 to 07 September 2021) of lockdown (representing stay-in-place orders described as being Alert Levels 3 and 4 in NZ) and times where COVID-19 restrictions eased (Alert Levels 1 and 2) [61]. The primary mode of mental health service delivery during these lockdown periods was via telehealth. These periods of lockdown are coloured in yellow and red in the graphs below.

**Figure 3:**
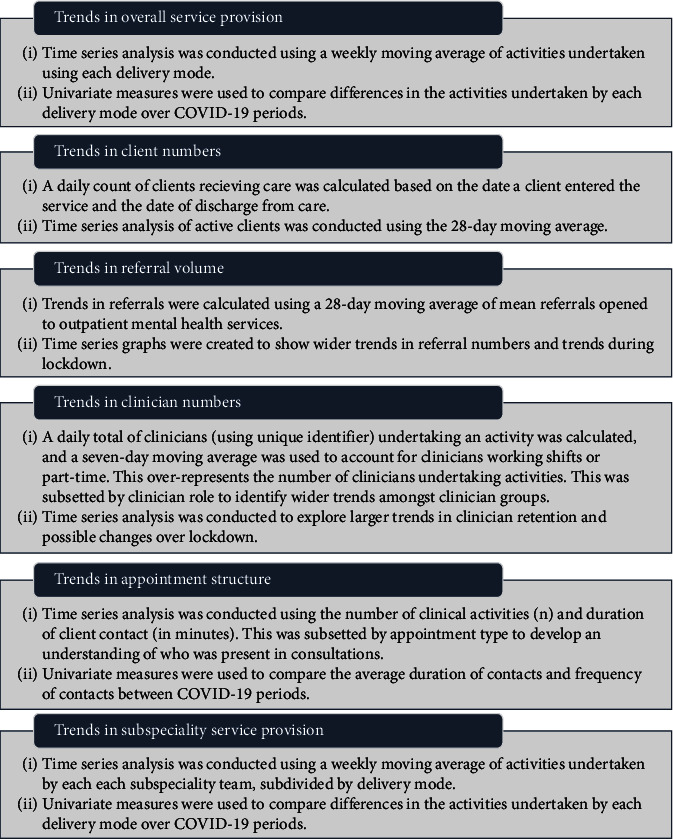
Analysis of key trends.

RStudio(×64 Version 4.1.2, Posit Software) was used for data cleaning and analysis. Data cleaning is outlined in Werkmeister et al. [48] but generally involved a process of (1) checking for consistency; (2) removing unnecessary entries tied to redacted information, inpatient, external, and operational teams, and nonmental health clinical positions; (3) standardising fields with spelling variations; (4) defining assumptions related to work days, additional duties, work activities, and COVID-19 alert levels; and (5) categorising data based on clinical team specialty, activity type, service delivery mode, and various client demographic characteristics. Time series analysis of outpatient activity data was conducted to explore trends in key aspects of service delivery, outlined in Figure 3.

### 2.6. Ethical Issues/Statement

Ethics approval was obtained from the Victoria University of Wellington Human Ethics Committee (#28808), and research endorsement was provided by the regional Research Advisory Group (Māori) (#765). Written informed consent was obtained from all participants prior to interview participation, and all participants were informed of B.J.W.'s clinical background. Access to quantitative data was governed by a memorandum of understanding between the researchers and the mental health service.

## 3. Results

**Figure 4:**
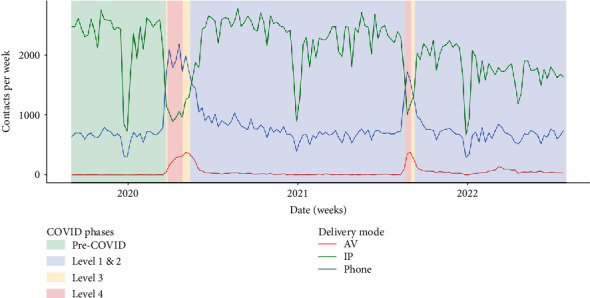
Service delivery by mode. AV = audiovisual, IP = in-person, Phone = telephone. December–January drop in referral numbers due to Christmas/New Year public holidays.

Leading up to the first COVID-19 lockdown in 2020, there was negligible prior use of AV delivery modes (Figure 4). At this time, mental health clinicians provided approximately three-quarters of their services in-person and one-quarter over the telephone. Participants described moving rapidly away from in-person services to telephone and AV modes to minimise the risk of COVID-19 exposure, due to anxiety about providing in-person care during the pandemic. This experience was mirrored in the administrative data (Figure 4).

*"If I'm carrying the virus around, if someone else is probably carrying the virus around, if we can do things by telehealth, that's going to be a lot safer." (P12)*

Participants reported that clients preferred to communicate with their clinician by telephone over lockdown, which is supported by the disproportional increase in telephone contact compared with AV modes (Figure 4). They thought that clients were uncomfortable bringing clinicians (virtually) into their personal space and that face-to-face contact was confrontational for some clients. However, some participants attributed this preference to avoidance behaviour by anxious clients and felt that telephone contact could be countertherapeutic.

*"There were one or two that really, really liked it, because they were anxious, and it gave a big break from anxiety. The outcomes have looked like they were better, but then I think it was actually just avoidance." (P21)*

Despite still being in the midst of the pandemic, following periods of lockdown, mental health services withdrew from AV modes and returned to pre-COVID-19 service provision modes. Interview participants proposed that the uptake of telehealth and return to pre-COVID-19 service provision was driven by a number of factors. This paper focuses on two overarching themes that arose during interviews.

1. *Wider trends in service provision that impacted telehealth uptake*, which refers to changes in client and clinician numbers, and the change in consultations over the study period; and
2. *Environmental factors influencing clinician use of telehealth*, which explore how the health system, social expectations, and clinical settings influenced clinician decisions to use telehealth.

### 3.1. Wider Trends in Mental Health Service Provision

Our analysis identified three significant trends that negatively impacted the regions' abilities to provide mental health services *increasing volume of clients receiving care*, *variation in new referrals*, and *inadequate staffing levels*. Each of these is considered here. Participants proposed that these factors, in the context of COVID-19 lockdown, forced clinicians to change the *structure of clinical appointments* and the type of care they provided.

#### 3.1.1. Increasing Volume of Clients Receiving Care

Participants described gradually increasing service demands, due to growing caseloads, more complex mental health needs, and clients requiring longer periods of outpatient treatment. Participants felt overwhelmed by their caseload and felt that the health system needed to reassess what services should be offered to clients, due to resource limitations. Participants further proposed reviewing criteria for accessing outpatient mental health services to create more manageable caseloads. They suggested that increasing caseloads and responsibility for managing client risks (suicide, violence, and poor self-care) resulted in burnout and poor clinician retention. Participants felt that telephone and AV modes should be part of long-term solutions to meet increasing demand, as this improved service flexibility and efficiency.

*"We're busier than before the lockdown… I've never had a caseload higher than the one I've got today… My caseload, half of it is chronic long-term women living independently on their own often. The other half was high risk people with borderline personality disorder… [Zoom] was easier for a lot of people of course, some of them [clients] wanted more." (P18)*

**Figure 5:**
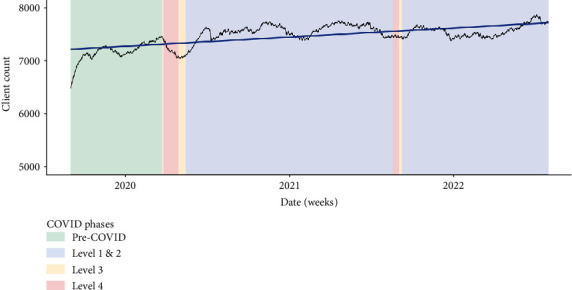
Clients receiving outpatient mental health treatment.

This theme is supported by quantitative findings (Figure 5); there is an overall upward trend in clients receiving outpatient mental healthcare in the study regions between September 2019 and August 2022. There is a small decrease in clients during lockdown, which reflected lower referral rates during lockdowns as discussed in the next section.

#### 3.1.2. Variation in New Referrals

Participants perceived that they received fewer referrals during the COVID-19 lockdowns. They proposed that clients developed agency to manage their care needs and developed closer bonds with family or peers, through a collective sense of hardship caused by COVID-19. They further suggested that clients were less likely to access mental health support due to improved supports and normalisation of their hardship.

*"The whole world was in this hyper-anxious state, meant that their anxiety was more normal, like other people were going through what they normally go through, so it was somehow normalising for them." (P2)*

Participants feared that referrals were artificially low during lockdown as clients were unable to see their general practitioner promptly to access referral during lockdown. They were concerned that referrals would increase following lockdown due to deferred presentations to general practice and delayed consequences of COVID-19 lockdown (such as financial hardship and job loss). Clinicians felt that the regional mental health system was ill-equipped to manage the potential influx in referrals as the system was overburdened.

*"It was actually coming out of lockdown, immediately after level three that we found we started to have an influx of people distressed. It was almost like during level four, people just held it together." (P3)*

**Figure 6:**
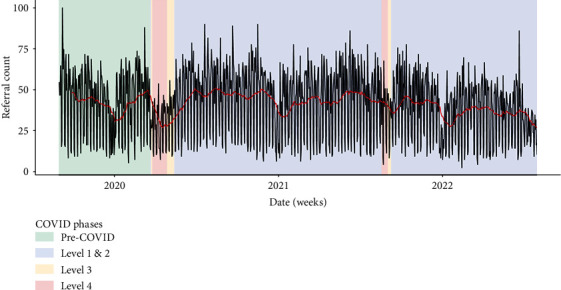
New referrals to outpatient mental health services. December–January drop in referral numbers due to Christmas/New Year public holidays.

These claims are partially supported by quantitative findings, which show considerable variation in referral patterns but also clearly reduced referrals during lockdown and an increase in referrals leading up to and following the first 2020 lockdown (Figure 6). However, referrals have since returned to pre-COVID-19 baselines.

Participants felt that improved liaison with general practitioners and other mental health clinicians through offering AV consultations to clients attending a general practice could improve care management in primary care settings and consequently influence referral processes. Participants suggested that providing early specialist advice to general practitioners could reduce the need for referral to specialist mental health services. However, they also suggested that mental health services were unable to provide effective AV consultations, and general practice liaison services were not designed to conduct assessments in-person.

*"Not all the referrals we get are actually in crisis… If somebody says, ‘oh God, I'm fed up with this, I'm going to end my life', without really pursuing that statement any further, people just go to crisis team and then you [the client] go and be waiting in ED for hours, whereas if I rang you, if I Zoomed you and talked to you and tried to pursue what that statement is, most of the time you [the general practitioner] are just frustrated. And I can guide you." (P23)*

#### 3.1.3. Inadequate Staffing Levels

Participants raised concerns about inadequate staffing across outpatient mental health services due to resignations and clinician illness. Participants found that due to shortages of psychologists, nurses, and doctors, their teams struggled to provide psychological therapy and diagnostic assessments and manage complex clients. Due to a moral duty to provide care for their clients, participants reluctantly agreed to increase caseloads, when colleagues were unwell or had left the service. Furthermore, participants felt they could not take a break, as their caseload would remain unchanged and work would accumulate in their absence. Participants felt that this increased clinician stress and contributed to burnout within mental health teams.

*"Burnout is so intense, it's so high. I know what that looks like… Sometimes you cannot take that stress [high caseload] away." (P11)*

**Figure 7:**
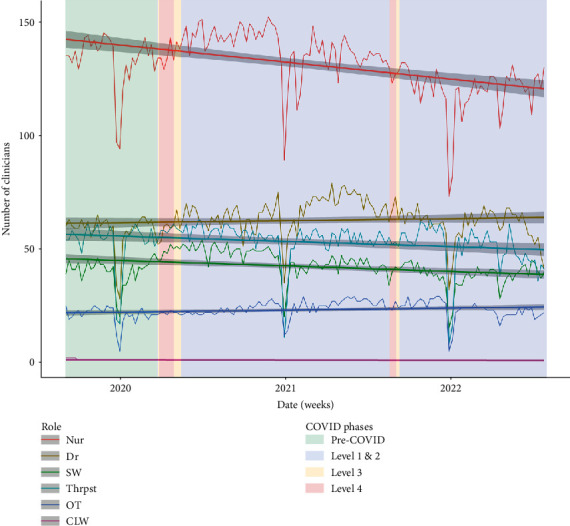
Weekly average of practicing clinicians by role. Dr = doctor, Nur = nurse, OT = occupational therapist, SW = social worker, Thrpst = therapist. Drop in clinician numbers over December–January due to Christmas/New Year public holiday leave.

Participant concerns surrounding staffing are supported by quantitative findings.  Figure 7 lays out the study regions' mental health workforce composition. There was a significant loss of nursing staff and gradual decreases in other clinician groups across the study period. Notably, in 2022, there were steeper declines in doctors and therapists. There was less variation in social worker, occupational therapist, and cultural liaison roles. However, due to the small base number of these clinicians, the loss of one clinician may have prevented teams from providing multidisciplinary care.

#### 3.1.4. Structure of Clinical Appointments

Participants described providing shorter more frequent appointments with clients over lockdown to respond to client needs. They moved away from formalised therapeutic interventions (such as cognitive behavioural therapy) that progressed treatment to supportive contacts (regular check-ins, validation, skill coaching, and supportive discussions with family). Participants found that supportive contacts took up to 20 min to conduct, compared with therapeutic interventions that often followed a formal structure, requiring 40–60 min to complete. Furthermore, participants described postponing therapeutic interventions until lockdown ended, as they were not familiar with using AV modes to conduct these interventions. Participants were concerned that postponing interventions during lockdown could delay client recovery or lead to deteriorations in mental state.

*"Skill coaching, really those basic things. It was really hard to do any cognitive therapy. It was around doing more behavioural stuff, which in some ways maybe was the right thing to do with lockdown. And checking in with them, they would say it was helpful, but it did not feel as effective as face-to-face. They were fine putting off therapy but, in my mind, I could see things slipping back." (P15)*

**Figure 8:**
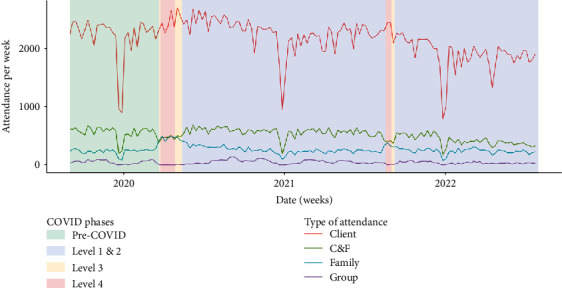
Weekly activities by type of attendance. Client = contact with client, C&F = client and family contact, Family = family only contact, Group = group activity. Drop in December–January attendance due to Christmas/New Year public holidays.

Trends in client attendance (Figure 8) illustrate increased frequency of client contact during lockdown, which supports participant claims. Furthermore, there is a wider trend of decreasing client appointments across the study period that reflects the decline in mental health clinician numbers (Figure 7), suggestive of workload saturation. In the context of increasing client numbers (Figure 5), this further supports participant experiences of overwhelming workloads attributed to inadequate staffing.

**Figure 9:**
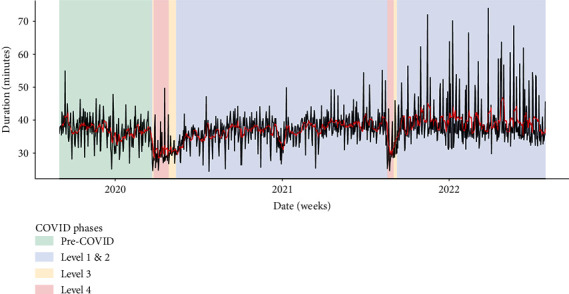
Average activity duration.

Trends in activity duration (Figure 9) illustrate a drop in the average duration of clinical activities during lockdown, which supports participant assertions.

Participants indicated that their clients' families required additional support during lockdown due to increased anxiety and social isolation. This is seen in Figure 8, which shows an increase in family contact during Levels 3 and 4 lockdowns. This gradually returned to baseline following return to Alert Levels 1 and 2. There was a small decrease in joint meetings with client and family over lockdown, which participants attributed to a lack of experience with group videoconferencing and clients being uncomfortable talking with family present.

*"It wasn't just with our whaiora [Māori clients], it was their whole family. There were a few times that I'd be passed around on the phone. Have a talk with mum then passed over to have a talk with big sister then little sister then the whaiora, like how everybody was feeling." (P9)*

### 3.2. Environmental Factors Influencing Telehealth Use

Participants found that the environment in which clinicians operated influenced their readiness to implement and sustain telehealth use. They identified four driving forces within their mental health teams: *culture of telehealth use*, *policy and guideline support*, *subspeciality team demands*, and *technical support*.

#### 3.2.1. Culture of Telehealth Use

Participants felt that the culture of service provision in the study regions did not support telehealth use. They thought that this was rooted in the historical absence of using AV delivery modes and limited appetite for change. They identified that a lack of training, awareness, and normalisation of AV modes in the study region contributed to a perception that telehealth was inferior to in-person service delivery. Participants thought that this contributed to clinician reluctance to use AV modes during lockdown and subsequent rapid retreats following lockdown.

*"Inevitably, it's here, telehealth is here and it's useful in some situations more than others… It's a little bit of a culture change that is needed as well for it to really be embraced as something that is not inferior to face-to-face." (P2)*

Participants thought that there was a lack of champions and communication with service leadership to support implementation of telehealth and drive culture change. They found that leadership did not have time to plan how to deliver telehealth, due to rapid transition to lockdown, resulting in poor coordination and implementation. Clinicians who were interested in supporting implementation (as champions) felt that they did not have clear direction from managers, resulting in clinicians providing ad hoc support to their team.

*"You have to keep the eye on the ball to make sure that there is progress… I've pulled back over the last couple of years hoping that the members of the team would step up, because there's some real experience here… You really have to keep your foot on the pedal to the finishing line."(P27)*

#### 3.2.2. Policy and Guideline Support

Participants found that there were limited guidelines and procedures to support the implementation and ongoing delivery of telehealth. They wanted procedures detailing how to set up AV clinics, as clinicians were unfamiliar with telehealth care delivery. They also wanted guidelines to manage caseload size and outline clinical activities that could be provided using telehealth.

*"I said right at the start is not having clear guidelines. I do not remember anyone saying you do not have to try to keep up your client contact as much as you would when you are in the office, when you are out of the office... I was still frantically trying to fit people in." (P1)*

Participants faced unique challenges managing privacy and confidentiality in AV consultations. They found that individuals could virtually intrude, or people in the client's household could inadvertently walk into AV appointments and breach client confidentiality. Technically knowledgeable participants questioned if AV applications were vulnerable to data breaches due to inadequate encryption. Participants felt that guidelines on managing privacy in AV consultation and policy on data storage and encryption could improve clinician confidence in navigating these issues.

*"How secure is Zoom? How secure are phone calls? I do not know. I do not think we have had a lot of guidance around the use of [telehealth]. I do not think there's any policies that I'm aware of around the use of telehealth." (P3)*

Participants feared that clients could breach clinician privacy by recording conversations and using extracts out of context on social media or to make fraudulent claims. Technically, knowledgeable participants raised concern that a service provider (third-party provider) could access private consultations and medical information stored by the provider. This raised questions about ownership of medical information and harm related to breaches of client and clinician privacy. Participants wanted an updated policy on ownership, storage, and accessing of medical information that included AV consultations, to protect both clinician and client privacy.

*"The guidelines are [now] saying every psychiatric contact is avoided to be recorded… It's then a question about who is owning this video, because patient information usually, so file notes, are not owned by the patient, and need a certain access and revision before they can actually be seen, so it's a legal issue. I think, it makes you feel naked." (P19)*

Participants were unsure if AV assessments met legal requirements for assessments under the Mental Health (Compulsory Assessment and Treatment) Act 1992 [62]. The Mental Health Act provides governing legislation in situations where mental illness poses a threat to the client or the public. Compulsory treatment orders allow clinicians to enforce mental health treatment without client consent, while granting the client specific rights while undergoing treatment. Participants feared that clients would be discharged from the order if they were not seen in-person, as AV modes were not considered face-to-face assessment under this legislation. Participants were generally unaware that Mental Health Act legislation was temporarily expanded to allow AV assessments during the pandemic [62]. They identified that guidelines (on using AV modes) and local policy (endorsing the use of AV modes) would increase their confidence in providing remote Mental Health Act assessments.

*"Is [AV consultation] legally a face-to-face assessment and with that, was that sufficient to start the Mental Health Act. That's a very important question." (P19)*

#### 3.2.3. Subspeciality Team Considerations

Participants described that subspeciality outpatient teams operated in nuanced ways, contributing to differential uptake of AV and telephone delivery modes. Participants found that AV modes were not suited for managing clients in crisis settings, as the presentations were by their nature more severe mental illness. They explained that clients in crisis were more likely to have impaired capacity and greater risk of suicide or violence. Clinicians were often uncomfortable managing these situations remotely, due to the lack of AV experience, limited resources to contain risk in the community, and concern over the consequences of unmanaged risk (suicide or homicide). They felt that in-person deescalation of risk was more effective. Participants also acknowledged that clinicians could be assaulted during in-person contact and police could be requested in some cases to deescalate and transport clients to places of safety.

*"The really unwell we saw face-to-face. We met either in hospital or respite." (P19)*

Participants working in community, alcohol, and drug subspecialities found it difficult to monitor substance addiction via AV or telephone modes. Participants found it difficult to identify physical features of ongoing substance misuse, such as observing needle track marks (in intravenous drug use), smelling alcohol or cannabis residue, and conducting drug tests (urine) or alcohol breath tests. Participants felt that these observations were required to determine the level of ongoing substance use, due to the perceived limited fullness of client disclosure.

*"If part of your job is checking peoples' arms for track marks, then you cannot do that effectively. If part of your job is getting a urine drug screen, then you cannot do that. I fairly regularly get the UDS [Urine Drug Screen] and breathalyse people, I could not do that. At one point, I did wonder whether somebody was actually smoking a joint when I was talking to them [on the telephone]." (P2)*

Participants working in child and adolescent (CAMHS) teams found parents of young clients preferred using videoconferencing to take part in reviews. Participants working in older persons' mental health services found that carers preferred 'group AV contact' when taking part in appointments. Participants thought that this may reflect difficulties engaging multiple people on a telephone call and familiarity with group AV contact.

*"Majority of them, especially the ones over thirteen would rather do phone calling… There was a lot of self-consciousness or anxiety around doing that, so a bit of avoidance, whereas parents were quite happy to Zoom." (P13)*

**Figure 10:**
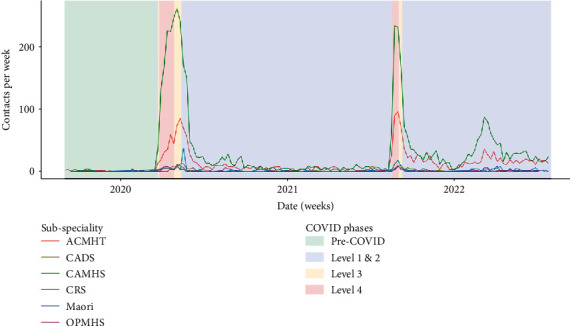
Audiovisual use by subspeciality. ACMHT = adult community mental health teams, CADS = community alcohol and drug services, CAMHS = child and adolescent mental health services, CRS = crisis resolution service, Māori = Māori mental health services (child and adult), OPMHS = older person mental health services.

Quantitative findings (Figures 4 and 10) support these assertions. CAMHS had the highest uptake of AV delivery modes, followed by adult services (ACMHT). There was little AV use by addiction service (CADS), crisis resolution service (CRS), and Māori mental health services.

**Figure 11:**
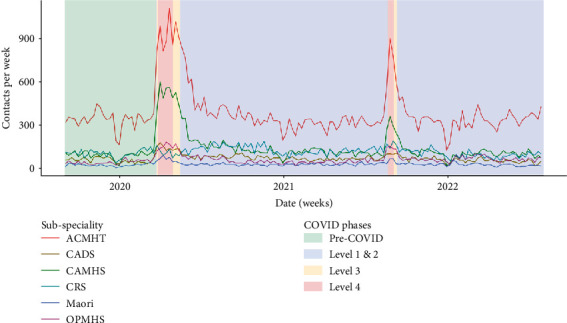
Telephone use by subspeciality. ACMHT = adult community mental health teams, CADS = community alcohol and drug services, CAMHS = child and adolescent mental health services, CRS = crisis resolution service, Māori = Māori mental health services (child and adult), OPMHS = older person mental health services.

ACMHT and CAMHS teams had the largest uptake of telephone contact during periods of lockdown (Figure 11). CRS clinicians had the least change in telephone contacts, despite participants reporting a dramatic increase in telephone consultation during lockdown.

#### 3.2.4. Technical Support

Participants used the term technical support to describe support provided by Information and Communication Technology (ICT) teams to set up telehealth software and hardware, troubleshoot technical issues during appointments, and set up telehealth clinics. Participants unfamiliar with AV technology felt they required technical support. At times, these people struggled to access technical support for real-time troubleshooting, forcing them to cancel AV appointments and revert to telephone or in-person appointments. Conversely, participants accessing timely technical support were more confident in continuing AV modes of service provision beyond times of lockdown.

*"For my colleagues who aren't so tech savvy, that was difficult. But my understanding is they did get IT [information technology] support… you could log ICT tickets for problems." (P1)*

Participants experienced disparate access to technical support during lockdown due to perceived prioritisation of medical, surgical, and hospital-based health services. They felt that centralised support services lacked capacity to meet demand due to limited resourcing and planning. Furthermore, during lockdown, travel restrictions prevented ICT personnel from visiting outpatient clinics (not located on hospital campus), removing the option of supporting clinicians in-person. Participants preferred in-person technical support due to poor experiences with remote technical support and limited ability to troubleshoot hardware problems remotely. Participants identified that additional resources to develop existing digital infrastructure and development of regional ICT teams could improve timely access to in-person technical support.

*"Some basic IT [information technology] support for the team as a whole and individually, like a half hour Zoom is on from IT for all of us to be like what is your system, what have you got, this is what you need to set up. Again, I think we did a good job of helping each other out with that but there's disparities there." (P25)*

## 4. Discussion

Telehealth has been used effectively internationally to improve access to healthcare in a number of mental health settings [24–26], including underresourced and geographically isolated areas [63–66]. This study builds on research into mental health service changes, telehealth implementation, and health service responses to COVID-19 by drawing together interviews with clinicians and almost 3 years of activity data. In so doing, it informs postpandemic decision-making. During the COVID-19 lockdowns in NZ, mental health services were forced to adapt to remote telehealth provision. However, the mental health system was not set up to support implementation of telehealth, due to increasing client demand, limited staffing, incompatible culture of service provision, lack of policy, limited technical support for clinicians, and nuanced drivers within subspeciality services. These system drivers hindered implementation and ongoing use of telehealth in the study region.

This study reflects national [67] and international trends [68] in mental health service demand and consequently highlights the impact of national trends in mental health workforce shortages [3, 69]. Mental health staff shortages and illness over lockdown posed a significant barrier to accessing services internationally [70]. Clinicians in our study felt that mental health services were not supported with policy or telehealth leadership during the COVID-19 lockdown, leading to inadequate resourcing and organisational direction. The mismatch identified in our analysis of activity data between staffing and service demand raises concerns about workforce sizing and the threshold for referral to specialist mental health services, to align client access with service capacity in resource-constrained environments. In turn, this stresses the need to consider how relief mental health workforce pools could be introduced to support times of increasing workforce pressure, suggestions similarly have been proposed in resource-constrained areas [71] and within groups in the mental health workforce [6].

Mental health clinicians are at risk of burnout [72, 73], which was exacerbated by a range of stressors over lockdown [18, 74, 75]. Our previous work highlighted that clinicians felt that they were at risk of burnout, due to working beyond their viable capacity [18]. The present study emphasises concerns of poor workforce retention and increasing demand on services, which reflects clinician experiences of working beyond capacity and burnout potentially contributing to poor retention. Notably, while our analysis of the nursing workforce points to significant long-term staffing issues, problems among doctors and therapists appear to have worsened since 2022.

Our present work has identified an urgent requirement to support clinician retention and reduce burnout through workforce planning. Such planning should include adequate workforce sizing and projections for population growth, relief pool of clinicians to cover clinicians taking annual leave, encouraging clinicians to take allocated leave, and, more importantly, putting processes in place to ensure continuity of support for clinical teams facing continuous low staffing levels. Formalised support could include collegial supervision [76, 77] or counselling for clinicians [78]. Understanding workforce pressures and requirements for improved support is timely, due to the upcoming mental health service redesign in NZ [49] as workforce retention and clinician wellbeing are fundamental to maintaining a health service.

COVID-19 and lockdowns had a significant psychological impact internationally [79, 80] and on the NZ population [81–84]. The observed increase in psychological distress did not translate into an increase in referrals over lockdown, suggesting barriers to referral arose due to COVID-19 restrictions. Potential barriers identified by participants in our study were the reduced availability of referrers (general practice and emergency services) and poor communication between primary care and secondary healthcare providers, which reflects emerging research into the interface between primary care and specialist mental health services [70, 85].

This study supports extant literature suggesting that telehealth could be used to address mismatches in supply and demand for mental health services [86–88]. Participants in this study identified that telehealth could be used to provide assessment and early specialist advice in the primary care setting. This could support primary care practitioners to treat more complex clients and reduce referrals to specialist mental health services for intensive treatment. Notably, variation identified in our study, particularly in new referrals (Figure 6) and average activity duration (Figure 9), points to issues that may exist in the ability to plan mental health service provision, further emphasising the need for pragmatic options to support a workforce struggling with providing services.

However, following on from our prior work [17, 18, 48], the potential for addressing issues with supply and demand for mental health services should be considered alongside the potential for digital exclusion [46, 89, 90]. Digital exclusion can exacerbate inequities in healthcare access for vulnerable client groups that often have sociodemographic issues [46]. This may be particularly true for the NZ Indigenous Māori populations who are at increased risk of digital exclusion [91–94] and can often struggle to engage with the healthcare system [95–97]. Our research emphasises the limitations in policy and guidelines, training and technical support, and client–provider relationships, which are all factors potentially limiting digital health equity [98].

Organisational culture is important for successful adoption of telehealth [99, 100]. This study found that the organisational culture within the study region did not support telehealth provision and clinicians did not accept telehealth as a suitable alternative to in-person care delivery. This study identified that telehealth promotion, coordination with managers, service planning, and change agents (service leadership and champions) are required to address cultures that do not support telehealth innovation, in line with international literature on implementing telehealth [101]. Future research into whether this cultural barrier is due to a perception of telehealth ineffectiveness or because of changes in the structure of clinical appointments delivered via telehealth modes (towards supportive contact) would be timely.

This study identified the need for guidelines to set up telehealth clinics and policy to address potential privacy concerns relating to data storage and ownership. This follows on from our prior work [17, 18, 48], which highlighted concerns around maintaining clinician and client privacy in consultations. Our work also reflects emerging literature on telehealth implementation during the pandemic, which emphasises demand for policy to define appropriate AV application uses and data storage security requirements [87, 102, 103]. A large study of telehealth policy implementation (across England, Wales, Scotland, and Northern Ireland) [104] found that clear policy facilitated interest in telehealth, reduced professional resistance to uptake, and could address some cultural barriers to implementing telehealth. The implementation process also highlighted significant variability in preparedness across the different services including issues of resourcing, staffing, and digital exclusion.

While there is a lack of policy driven by NZ public mental health service leadership and management, there are existing policies and guidelines endorsed by clinician professional organisations, such as the Royal Australian and New Zealand College of Psychiatrists [105]. This includes minimum standards for hardware, data security policies, and recommended applications for service delivery. These could be used as a basis for implementing standardised national telehealth policy and guidelines to support mental health clinicians. Service providers should engage with clinicians to review existing telehealth guidelines and develop policy to support telehealth implementation.

Participants felt uncertain that AV assessments met the requirements of mental health compulsory assessment legislation. During lockdown, the Mental Health (Compulsory Assessment and Treatment) Act 1992 was amended to provide flexibility to use AV assessment in a limited capacity [62]. This engenders discussion around the appropriateness of AV delivery modes for inclusion in mental health legislation, as there is limited evidence (noninferiority trials) for using telehealth in crisis settings [106–109] and situations involving severe mental illness and risk (suicide or self-harm) [110], where this legislation is used. However, there is a slowly growing body of international literature that suggests that telehealth can have a role in managing severe mental illness [111, 112] and suicide risk [110, 113, 114] in crisis settings. This should be taken into consideration in the ongoing review of Mental Health Act legislation in NZ [115]. Such considerations should be taken alongside continuous monitoring of the effect implementing AV modes in usual business could have on decisions clinicians make to enforce compulsory treatment.

This study identified that subspeciality mental health teams had adapted their assessment and management approaches to suit the conditions and situations unique to their subspeciality. This created unique challenges for providing telehealth including group consultation with caregivers, avoidance behaviours, managing risk (in acutely unwell clients), and diagnoses that did not lend well to telehealth. There is emerging research into managing avoidance behaviours [116, 117], suicide risk [110, 113, 114], and substance abuse disorders [118, 119] via telehealth. However, further research is required as current literature is poorly generalisable, due to specific populations (veterans and rural) and conditions (mood disorders and personality disorders), making it difficult to apply to subspeciality outpatient settings.

This study identified clinician requirement for support to address technical challenges and useability issues. International literature on telehealth implementation [103, 120, 121] underlines the value of ICT support, improving technical infrastructure and integrating telehealth platforms with electronic health records, to make remote service provision a more viable option for clinicians and clients. There is a need for scalable information technology capabilities to respond to public health crisis (such as future pandemic) and accommodate future demand, so that clinician can access real-time support to provide services remotely. Postpandemic, this is particularly important given changes in client expectations caused by having experienced telehealth delivery modes.

### 4.1. Key Recommendations

Table 2 lays out key recommendations for clinicians and health system management.

### 4.2. Limitations and Additional Future Research

The dataset used in the quantitative component of this research is the only client activity data record for the study region. There were limitations in consistency of data collected as this dataset relied on clinicians and administrative staff recording appointment data accurately. In addition, the dataset did not contain the purpose or content of appointments, which limited inferences around client outcomes and clinician consultation approaches.

Future research should explore how the rapid transition to telehealth over lockdown influenced clinical outcomes and evaluate what has contributed to clinicians continued use of telehealth in business-as-usual practice. Part of this could also involve assessing the impact longer term use of telehealth may have on clinical outcomes and its use in specific populations. In turn, in the NZ context, further research is required into implementation and use of telehealth for Māori populations, who already experience health inequities and are at increased risk of digital exclusion, may lead to a better understanding of equity-related issues. While this study identifies inadequate technical support to support telehealth uptake, there is little evidence on the ongoing setup and running costs of a larger telehealth service. Economic evaluation that directly includes an assessment of equity [122] could support recommendations around future service planning and resource allocation to mental health teams when developing telehealth clinics.

## 5. Conclusions

Mental health services in the study region implemented telehealth out of necessity during the COVID-19 pandemic. However, telehealth operation rapidly reduced outside of lockdown due to environmental drivers that hindered its implementation and ongoing use. Telehealth has the potential to improve interfaces between primary care and specialist mental health services and be used as an alternative to in-person delivery modes. This study raises concerns around workforce retention and service growth within the study regions, which may be better managed by implementing complementary telehealth modes. This requires broader organisational changes, including strong leadership and champions to change the service provision culture, policy and guidelines to support clinicians, and improved ICT infrastructure and real-time technical support. These environmental factors should be considered in service planning across larger mental health services, to improve integration of telehealth into clinical practice and address the mismatch between service demand and capacity.

## Figures and Tables

**Table 1:** Reframed assertions for testing.

**Reframed assertions**
There was limited uptake of audiovisual (AV) services.
There was greater uptake of telephone than AV service delivery during lockdown.
There was differential uptake of AV services among subspecialty teams.
There was differential uptake of telephone services among subspecialty teams.
Demand for mental health services has been increasing.
During lockdown, new referrals to mental health services decreased.
Clinician numbers have been decreasing.
Appointment frequency for clients increased during lockdown.
Appointment duration decreased during lockdown.

**Table 2:** Key recommendations.

	**Clinicians**	**Health system managers**
Referrals	• Telehealth can be used to triage clients referred to mental health services. • Telehealth can be used to improve communication with referrer and provide early specialist advice.	• Discuss suitability of current referral criteria with clinicians. • Consider expanding mental health liaison services with primary care providers.

Staffing and burnout	• Discuss appropriate caseload sizing with team leaders, including considering whether some clients could be supported using telehealth. • Discuss working from home using telehealth to balance workload.	• Consider supporting the management of workforce shortages through clinicians working remotely using telehealth in hard to staff regions. • Offer flexibility of working from home by telehealth to support clinician wellbeing and therefore retention.

Culture	• Promote discussion on the use of telehealth in business-as-usual practice with colleagues. • Inform clients about what services are offered by telehealth.	• Create a multidisciplinary cross-specialty telehealth working group to develop telehealth training, grow champions, and build telehealth awareness to promote and support culture change and telehealth stewardship.

Policy and guidelines	• Clinicians and clients should not record (audio and/or visual) appointments, to avoid privacy breaches. • Introduce all participants taking part in telehealth consultation, to avoid breaches of confidentiality. • Clinicians offering telehealth should review their data management and consultation privacy practices to align with national standards.	• Create policy to support the use of telehealth, by outlining what telehealth can be used for, including interpretation of mental health legislation. • Create policy for addressing clinician privacy and data management. • Develop local or implement existing guidelines for clinicians to set up telehealth appointments and clinics. These guidelines should define differences in expectations around one-off appointments, compared to establishing complementary telehealth clinics.

Team considerations	• Engage in discussion with health system managers, to develop an understanding of the needs of subspeciality teams, and how this affects their ability to provide telehealth.	• Develop resources to support clinicians to tailor care delivery to unique client needs within these services. • Develop a working group with clinicians to discuss nuances of telehealth provision among subspecialities.

Technical support	• Identify and report technical issues to support software improvement.	• Invest in telehealth infrastructure and technical support. • Develop ICT plan to accommodate increasing demand and emerging technologies. • Consider reorganisation of ICT support with regional services that could provide in-person support.

## Data Availability

Data analysed are the properties of the relevant mental health services and are not publicly available. The Memorandum of Understanding governing data use prohibits public sharing of data.
